# Contrasting biological features in morphologically cryptic Mediterranean sponges

**DOI:** 10.7717/peerj.3490

**Published:** 2017-06-29

**Authors:** Leire Garate, Andrea Blanquer, Maria J. Uriz

**Affiliations:** Department of Marine Ecology, Centre d’Estudis Avançats de Blanes (CEAB-CSIC), Blanes, Girona, Spain

**Keywords:** Sponges, Survival, Life span, Growth, Fusions, Fissions, Hemimycale columella, Hemimycale mediterranea, Mediterranean sea

## Abstract

Sponges are key organisms in the marine benthos where they play essential roles in ecological processes such as creating new niches, competition for resources, and organic matter recycling. Despite the increasing number of taxonomical studies, many sponge species remain hidden, whether unnoticed or cryptic. The occurrence of cryptic species may confound ecological studies by underestimating biodiversity. In this study, we monitored photographically growth, fusions, fissions, and survival of two morphologically cryptic species *Hemimycale mediterranea* Uriz, Garate & Agell, 2017 and *H. columella* (Bowerbank, 1874). Additionally, we characterized the main environmental factors of the corresponding species habitats, trying to ascertain whether some abiotic factors were correlated with the distribution of these species. Sponge monitoring was performed monthly. Seawater samples were collected the same monitoring days in the vicinity of the target sponges. Results showed contrasting growth and survival patterns for each species: *H. mediterranea* totally disappeared after larval release while 64% of individuals of *H. columella* survived the entire two years we monitored. The species also differed in the number of fissions and fusions. These events were evenly distributed throughout the year in the *H. mediterranea* population but concentrated in cold months in *H. columella*. No measured environmental factor correlated with *H. mediterranea* growth rates, while temperature and dissolved organic nitrogen were negatively correlated with *H. columella* growth rates. The strong differences in depth distribution, survival, growth, fusions, and fissions found between these two cryptic species, highlights the importance of untangling cryptic species before ecological studies are performed in particular when these species share geographical distribution.

## Introduction

Sponges are worldwide-distributed invertebrates, inhabiting shallow to abyssal benthic habitats, at all latitudes (Reiswig, 1973; Uriz, Martin & Rosell, 1992; Hooper & Van Soest, 2002). They play a primary role in benthic assemblages by interacting in several ways with vegetal and animal neighbors (Wulff, 2006). Competition for space, provision of macro-and microhabitats for other organisms (Diaz & Rützler, 2001), organic matter recycling (De Goeij et al., 2013), and energy transfer from pelagic to benthic compartments (Gili & Coma, 1998) are some of the key functions that the sponges perform in marine ecosystems.

Sponge growth is still intriguing in many cases. Some species maintain the same size for decades (Teixidó, Pineda & Garrabou, 2009), and this trait depicts them as long-lived, slow growth organisms. However, when studied over shorter temporal scales (months to years), species that did not increase in size for years (Teixidó, Pineda & Garrabou, 2009) proved to be highly dynamic, with fast successive increases and decreases in size (Turon, Tarjuelo & Uriz, 1998). In fact, waxing and waning growth rates throughout the year have been documented for several encrusting Mediterranean sponges (Garrabou & Zabala, 2001; Blanquer, Uriz & Agell, 2008; De Caralt, Uriz & Wijffels, 2008), such that their ecological success appears to rely on keeping a colonized niche rather than on getting large.

*Hemimycale mediterranea* Uriz, Garate & Agell, 2017 was recently described as a new morphologically cryptic species with *H. columella* (Bowerbank, 1874) (Uriz, Garate & Agell, 2017). Genetic differences of what firstly though to be two *H. columella* populations, were higher than those expected to be considered intra-species variation. Phylogenetic and morphological analyses, subsequently performed, revealed that these two populations indeed belong to two cryptic species (Uriz, Garate & Agell, 2017). An *a posteriori* in deep search for phenotypic differences allowed us to record color tinge, diameter and rim high of the aerolate areas, and spicule size as the only species-specific traits. The type species of the genus, *H. columella,* seemed to remain stable for years (MJ Uriz, pers. obs., 2012), while the newly described, *H. mediterranea,* appeared to be an annual species, with population demise after release of larvae (Pérez-Porro, González & Uriz, 2012). Both species harbor abundant calcifying bacteria (Uriz et al., 2012; Garate, Blanquer & Uriz, 2015), which are through to protect the sponges from predation (Garate, Blanquer & Uriz, 2015). There were no signs of predation in the many sponges examined, which point to causes other than predation for explaining the contrasting species mortality patterns observed.

The two cryptic *Hemimycale* species (Uriz, Garate & Agell, 2017) share a geographical distribution thorough the Mediterranean but show contrasting depth preferences. *H. mediterranea* dwells on shallow waters (4–17 m deep) while *H. columella* is preferentially found from 25 to 60 m depth (Uriz, Garate & Agell, 2017). Thus, these species represented suitable targets to determine whether some depth-related environmental factors might account for contrasting ecological distribution of sponges. Temperature (Tanaka, 2002; Page et al., 2005; De Caralt, Uriz & Wijffels, 2008; Koopmans & Wijffels, 2008) and food availability, either particulate (Reiswig, 1973; Sebens, 1987; Riisgård et al., 1993; Ribes, Coma & Gili, 1999a; Ribes et al., 2005; Lesser, 2006; Koopmans & Wijffels, 2008; De Caralt, Uriz & Wijffels, 2008) or dissolved (Yahel et al., 2003; De Goeij et al., 2008; Mueller et al., 2014) are two of the main factors determining sponge growth. These two factors undergo notable seasonal variations in temperate seas such as the Mediterranean with potential limiting values for growth and survival of some filter-feeding species in summer (Coma et al., 2000).

The main objectives of this study were to assess the growth and survival patterns of these two genetically different but morphologically cryptic species, which differ in habitat preferences (Uriz, Garate & Agell, 2017) and to characterize the environmental factors of their respective habitats

## Material and Methods

### Study sites

*Hemimycale columella* and *Hemimycale mediterranea* were monitored in the NW Mediterranean: Iberian Peninsula, Catalan coasts (41°34′N, 2°33′E–41°42′N, 2°54′E). *H. mediterranea* dwelt on vertical shallow (12–17 m deep) rocky walls (hereafter, shallow habitats). *H. columella* grew on horizontal coralligenous assemblages (Casas-Güell et al., 2015) at 28–30 m of depth (deep habitats).

### Growth dynamics and survival

Both sponge populations were monthly monitored by SCUBA diving. A total of 24 randomly selected individuals of *H. mediteranea* and 27 individuals of *H. columella* were tagged using labels fixed with a two-component, water resistant resin (IVEGOR, SA) and photographed monthly. A SONY Cybershot digital camera was mounted on a custom-made structure consisting of a 20 × 14 cm frame fixed by a 30 cm long metallic support, to ensure the same focal distance and position during the entire monitoring period (Blanquer, Uriz & Agell, 2008). Estimates of survival, growth, regression, fissions, and fusions were derived from pictures taken monthly. The monitoring of *H. mediterranea* started in February 2012, when the individuals began to be conspicuous (i.e., image area greater than 1 cm^2^), and lasted until September 2012 when the species disappeared after release of larvae. Conversely, most labeled individuals of *H. columella* remained and were monitored from May 2012 to June 2014.

Monthly pictures of each individual were outlined and the area was calculated by using ImageJ software (Schneider, Rasband & Eliceiri, 2012). Both species mainly grow in two dimensions in the study sites (thinly encrusting growth shape) so that changes in area can be correlated to growth (increases) or shrinkage (decreases) (Garrabou & Zabala, 2001; Blanquer, Uriz & Agell, 2008). Monthly growth rates were derived from the formula:

\begin{eqnarray*}G=(({A}_{m}-{A}_{m-1})/{A}_{m-1})/t \end{eqnarray*}

where *A*_*m*_ is the sponge area at month *m*, *A*_*m*−1_ the sponge area of the previous month and *t* the months between two recorded data (Turon, Tarjuelo & Uriz, 1998); *t* equaled 1 in general (monthly growth data) but was 2 in the few cases (i.e., January and March 2013) when sea conditions prevented sampling in a given month. In these cases, growth rates were calculated between two consecutive data recordings.

Survival curves were derived from the number of monitoring months that an individual was recorded. For calculations, when two sponges fused, the resulting individual was considered as a new one and the preceding two that fused were considered as dead, so that the final individual pool decreased in one individual. When one sponge split in two or more clones, the individual was considered as dead and the resulting individuals were counted as new ones (De Caralt, Uriz & Wijffels, 2008).

### Fusion and fission events

The percentage of individuals of each species undergoing fissions or fusions along the monitoring period was also recorded. Whether the events were single, double, triple, or quadruple was noted. A single fusion event was the fusion of two sponge fragments between two observations, a double fusion event was recorded where three sponges fused, and so on. Similarly, when an individual split in two fragments, one fission event was recorded and when it was divided in three fragments between two observations, a double fission event was recorded (Blanquer, Uriz & Agell, 2008). When two or more sponges fused, they were treated as one individual for the following monitoring month.

### Environmental factors

Analyses of abiotic factors were performed monthly during the entire monitoring period (i.e., seven months for *H. mediterranea* location and ca. 24 months for *H. columella* location). Water samples (three 500 ml replicates) were collected in glass bottles by SCUBA diving in the vicinity of the tagged sponges and taken in the dark in a cooler (ca. 4 °C) to the laboratory. Once in the laboratory, 400 ml of water of each replicate were filtered through pre-combusted GF/F filters (450 °C for 4 h), using a baked glass filtration system, previously cleaned with a 10% HCl solution for 24 h. The filters were stored at −80 °C until the monitoring period finished and then they were analyzed for particulate organic carbon and nitrogen—POC and PON–. The filtrate was used for analyses of dissolved nutrients.

For POC and PON analysis, the frozen filters were dried at 60 °C during 24 h and then analyzed at Scientific and Technological Services of the University of Barcelona, using an elemental organic analyzer Thermo EA 1108 (Thermo Scientific, Milan, Italy) working in standard conditions as recommended by the supplier (i.e., helium flow at 120 ml/min, combustion furnace at 1,000 °C, chromatographic column oven at 60 °C, oxygen loop 10 ml at 100 kPa).

For dissolved organic (DOC) analyses, 10 ml of filtrate were collected in pre-combusted glass ampoules (450 °C for 24 h), heat-sealed and stored at 4 °C until the analyses were performed (every three months). For TN analyses, 15 ml of filtrate per replicate was collected in a Falcon tube, previously cleaned in an acid bath (10% HCl for 24 h). Dissolved inorganic nitrogen (DIN) was recorded at Operational Observatory of the Catalan Sea (CEAB-CSIC). Dissolved organic nitrogen (DON) was estimated by subtracting DIN from TN. DOC and TN were determined using high temperature catalytic oxidation method by a Shimadzu TOC-VCSH + ASI-V (ICM-CSIC).

Seawater temperature (*T*) was recorded every 6 h at the study sites using a StowAway TidbiT Temperature Data Logger, placed on the rocky bottoms of the respective habitats, close to the monitored sponges.

### Data analyses

Data did not comply with the normality (Shapiro–Wilk *W* test) and homoscedasticity (Cochran *C* test) assumptions of parametric tests and were rank-transformed. Comparisons of monthly sponge area and growth rates between the two sponge species were performed by multivariate analysis of variance (MANOVA). MANOVA does no require the dependent variables to be equally correlated as repeated-measures ANOVA does. The total number of replicates of both species used (*N* = 51) and the number of observation times (*K* = 25 months or *K* = 4 seasons) was adequate to warrant a high-test power for the analyses (Potvin, Lechowicz & Tardif, 1990). Post-hoc comparisons were performed by Newman–Keuls tests (Shesking, 1989). Mean growth rates and mean area changes during the entire period when both populations coexisted were compared by Newman–Keuls test. Monthly comparisons between each environmental factor at the two species sites were performed by two-way ANOVA.

Cross-correlation is a measure of similarity of two series as a function of the displacement of one relative to the other across time. Cross-correlation was performed between the growth rates and each measured environmental factor at a time window of one month. These analyses allow to determine the effect of the factor on the variable at the time lag = 0, as a simple correlation, or a different time lags (Weisstein, 2009). The analyses generate a histogram where two groups of bins are differentiated by left and right. The left group represents the negative times, when the factor growth rate fired prior to the environmental factor. The center or zero bin of the histogram accumulates the number of instances when the two values fired precisely together. The right group of bins accounts for the positive times when the growth values fired posterior to the environmental factor targeted (Weisstein, 2009).

Differences in the number and type (whether simple, double or multiple events) of fissions and fusions between sponge species did not require statistical analysis since no fissions or fusions occurred in *H. columella* during the months both species coexisted. Survival curves between the two species were compared using Wilcoxon-type test (Fox, 1993).

All analyses were performed with STATISTICA 6.0 (StatSoft, Inc., Tulsa, OK, USA).

## Results

### Growth and survival (Tables S1 and S2 )

**Figure 1:**
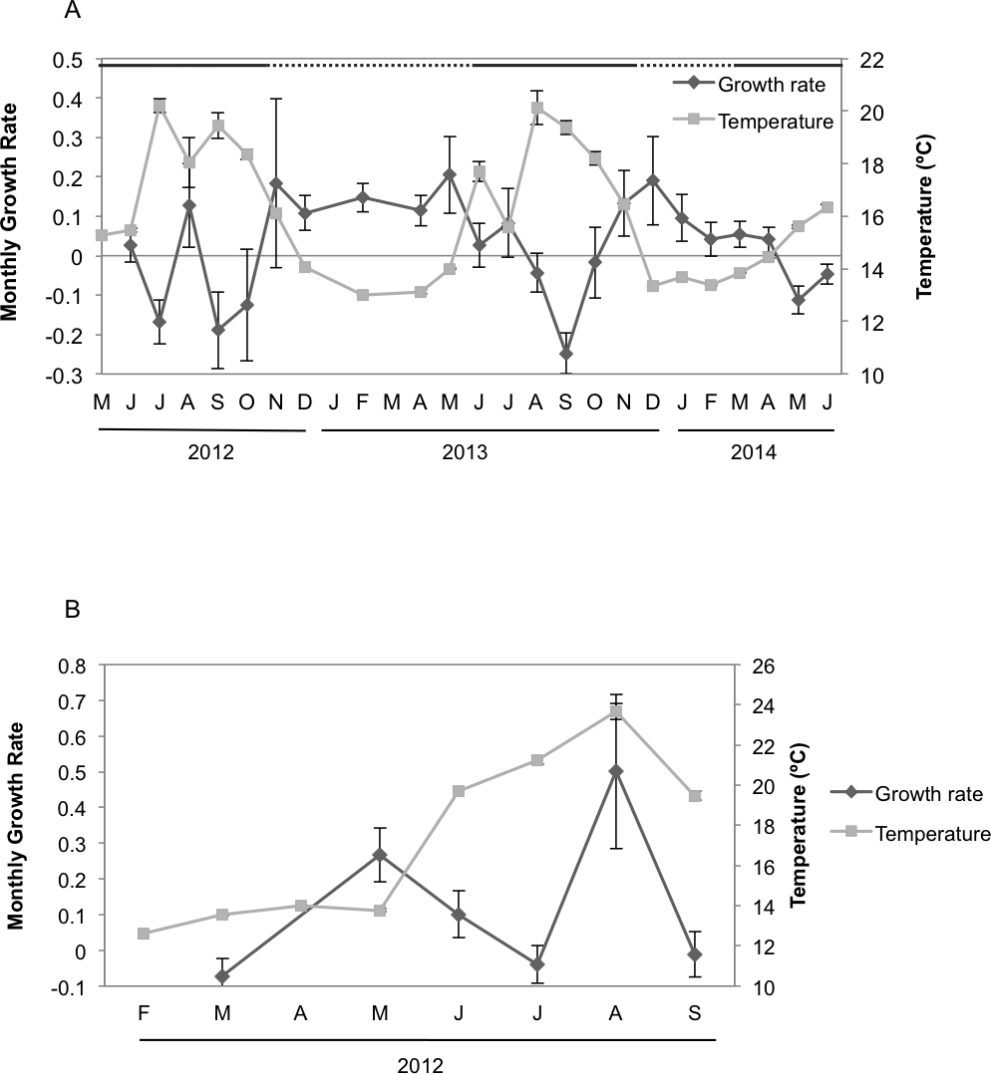
Mean growth rates of *H. columella* and *H. mediterranea*. (A) Monthly mean (±SE) growth rates of *H. columella*. (B) Monthly mean (±SE) growth rates of *H. mediterranea*. The light grey line represents monthly seawater temperature (°C) at both species habitats Continuous horizontal bars on the top of graphic A join months with no significant differences in growth rate (*p* < 0.0001).

**Figure 2:**
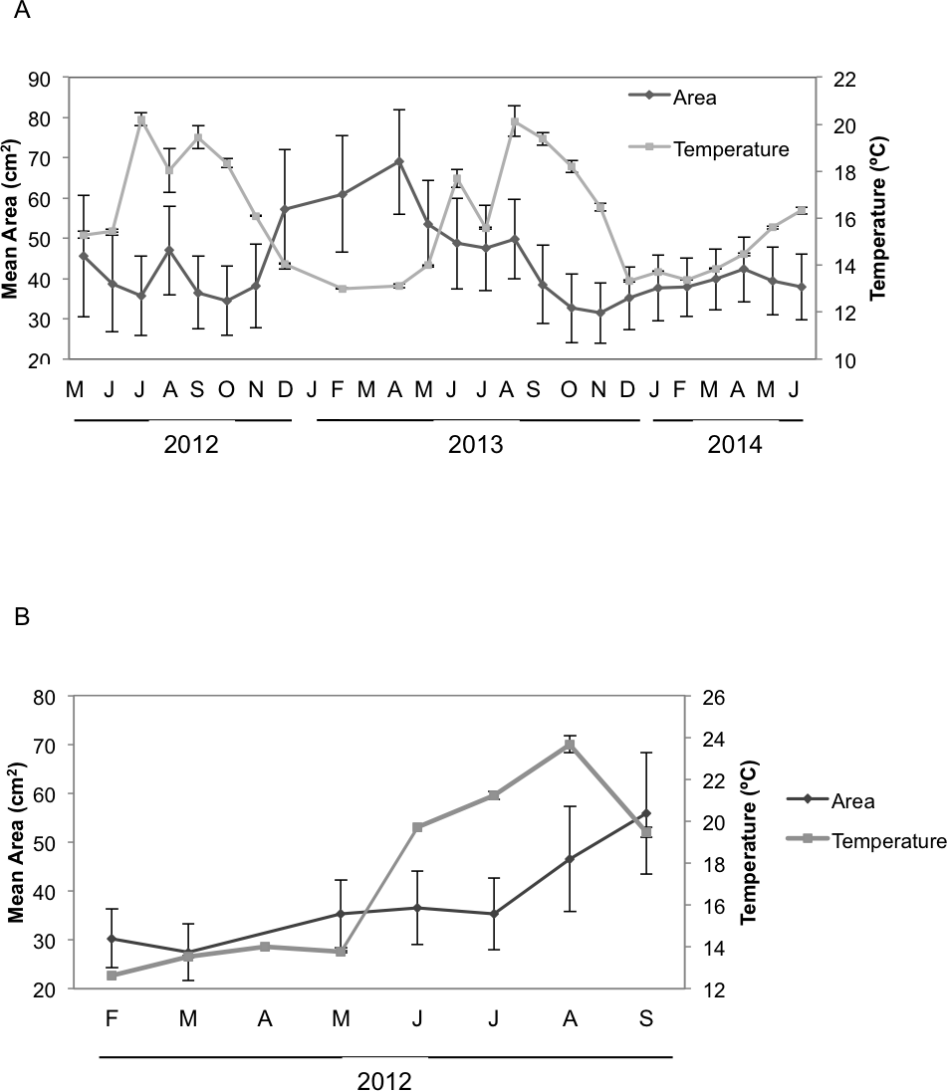
Mean areas of *H. columella* and *H. mediterranea*. (A) Monthly mean (±SE) area of *H. columella*. (B) Monthly mean (±SE) area of *H. mediterranea*. The light gray line represents monthly seawater temperature (°C) at both species habitats.

Seasonal sponge growth rates were significantly different (MANOVA *F* = 4.5, *p* < 0.001) for *H. columella*, showing higher values (post-hoc multiple comparisons *p* < 0.001) in winter (January–March) than in summer (July–September) (Fig. 1A). Conversely, no significant differences in seasonal growth rates were found for the *H. mediterranea* population (MANOVA *F* = 0.97, *p* = 0.422) (Fig. 1B). Mean areas showed no significant differences among seasons, although followed the same trend as growth rates (MANOVA; *F* = 0.34, *p* = 0.996 and *F* = 0.35, *p* = 0.998 for *H. columella* and *H. mediterranea*, respectively) (Fig. 2).

**Figure 3:**
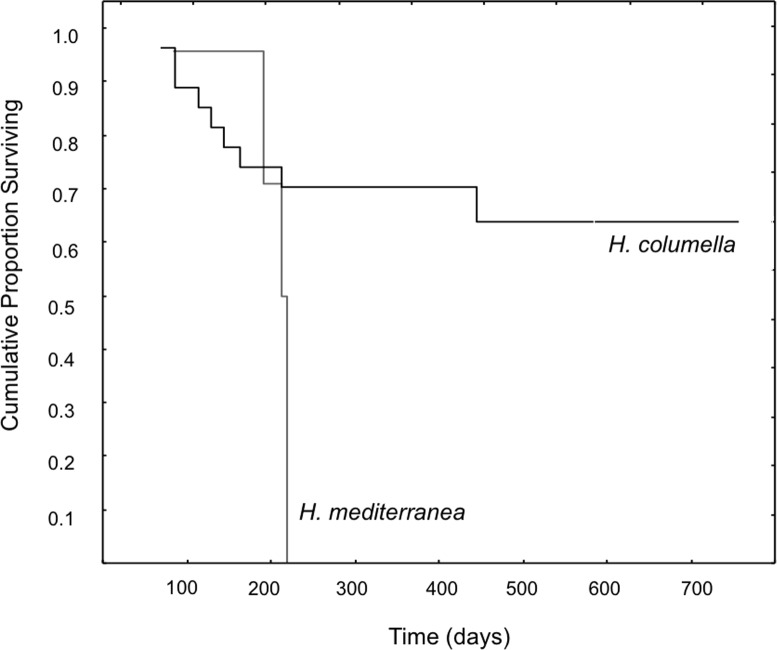
Survival curves of *H. columella* and *H. mediterranea* (Wilcoxon test, *p* < 0.005).

When we considered the entire period when both species coexisted, the final mean growth rates were significantly higher for *H. mediterranea* than for *H. columella* (Newman–Keuls test, *F* = 13.94, *p* < 0.001), which approached 0, as growth (increase in area) and shrinkage (decrease in area) were compensated along study months. However, differences in mean area between species during the same period were not significant (Newman–Keuls *F* = 0.21, *p* = 0.989). Survival curves were significantly different for both species (Wilcoxon test *p* < 0.005). While no one individual of *H. mediterranea* survived in shallow environments after seven months (i.e., after larval release), ca. 70% of the monitored individuals of *H. columella* survived in the deep environments during the same period and 64% survived at the end of two years of monitoring (Fig. 3).

### Fission and fusion events

**Figure 4:**
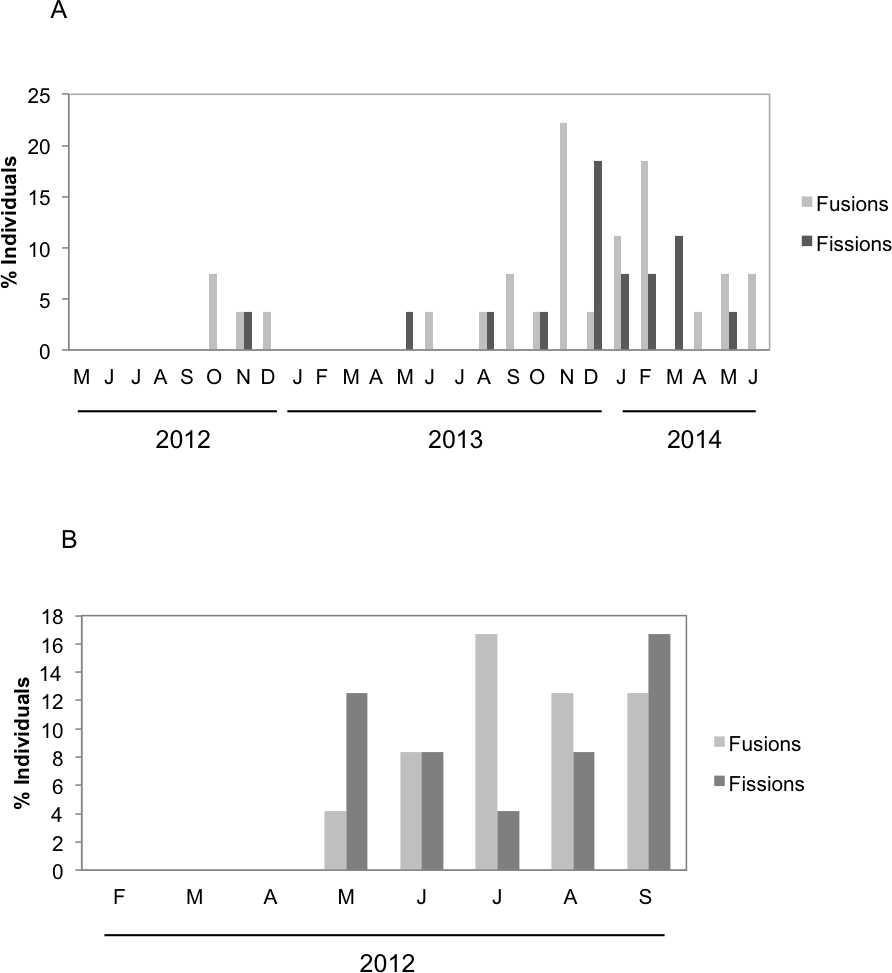
(A) Percentage of *H. columella* individuals experiencing either fission or fusion events. (B) Percentage of *H. mediterranea* individuals experiencing either fission or fusion events.

**Figure 5:**
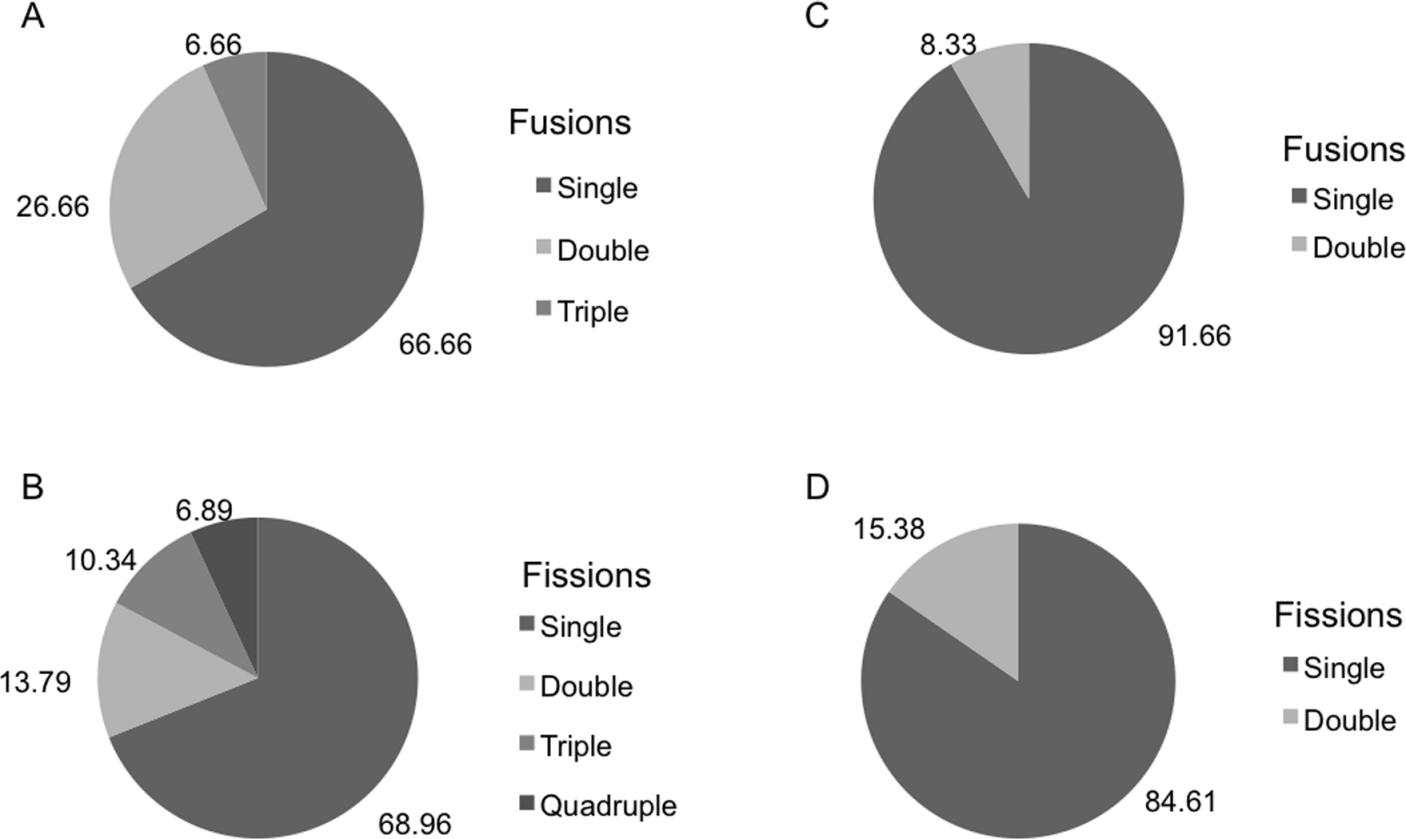
(A, B) Percentage of *H. columella* individuals showing from one to four fusions or fissions. (C, D) Percentage of * H. mediterranea* individuals showing single or double fusions and fissions. Single, double, and triple fission and fusion events.

There were significant differences in the number and type of fissions and fusions between the two species during the months both species coexisted (Fig. 4). Fusions of *H. mediterranea* increased from May to July and then slightly decreased in August–September (Fig. 4B). Fissions of *H. mediterranea* were mainly recorded in May and September after the end of the reproduction and prior to the populations death (Fig. 4B).Conversely, in *H. columella*, no fissions were recorded at the end of the reproduction period (October) but they occurred preferentially in winter (Fig. 4A). The number of fusions increased in autumn-winter of the second monitoring year to decrease in the following spring months (Fig. 4A). Moreover, fusion and fission events were single or double in *H. mediterranea*, while also triple fusions and quadruple fissions occurred in *H. columella* (Fig. 5).

### Environmental factors

All environmental factors analyzed (Tables S3 and S4 tables) varied significantly (ANOVA, *p* < 0.05) throughout the year in the habitats of both species, and all of them but PON (ANOVA, *F* = 0.016, *p* = 0.90) and *T* (ANOVA, *F* = 0.64, *p* = 0.59) were significantly different between shallow and deep habitats.

### Temperature (*T*)

The highest *T* values were detected from June to September in both habitats, corresponding to the Mediterranean summer. However, temperature reached up to 24 °C in shallows habitats but peaked 20 °C in deep habitats. The minimum *T* was similar (ca. 12.5 °C) at both depths in winter (February) (Fig. 1).

### Dissolved Organic Carbon (DOC)

**Figure 6:**
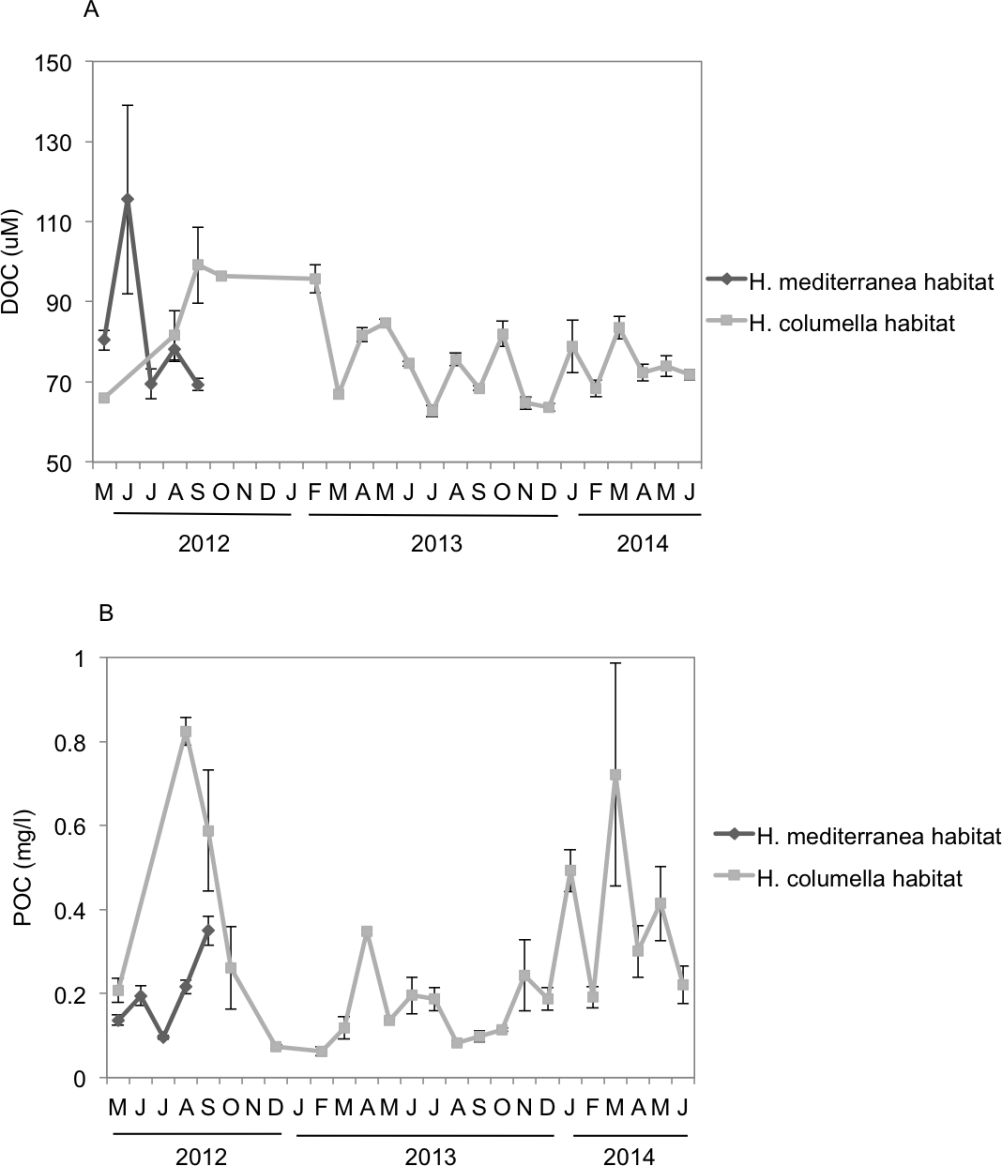
(A) Mean (±SE) concentration of Dissolved Organic Carbon (DOC). (B) Mean (±SE) concentration of Particulate Organic Carbon (POC) in the *H. columella* and *H. mediterranea* habitats, during the entire study period.

DOC showed a strong monthly variation at both depths. In the months when both species coexisted, significant differences in DOC concentration were found between depths (*p* < 0.05). DOC values were higher in shallow habitats during May and June (80–115 µM) than in the deep habitats, but the trend changed the following months. DOC concentrations (80–100 µM) in August and September were higher in deep habitats than in shallow habitats (Fig. 6A).

### Particulate Organic Carbon (POC)

In the months when both populations coexisted, the deep-water habitats were richer in POC than the shallow-water habitats (Two-way ANOVA, *p* < 0.05) (Fig. 6B). In this period, POC ranged from 0.1 mg/L in July to 0.35 mg/L in September (shallow habitats) and from 0.2 mg/L in May to 0.95 mg/L in July (deep habitats). In *H. columella* habitats, during the 21 months after *H. mediterranea* disappeared, POC showed two peaks in winter (one of 0.5 mg/L in January 2014, and the other of 0.75 mg/L in March 2014).

### Dissolved Organic Nitrogen (DON)

**Figure 7:**
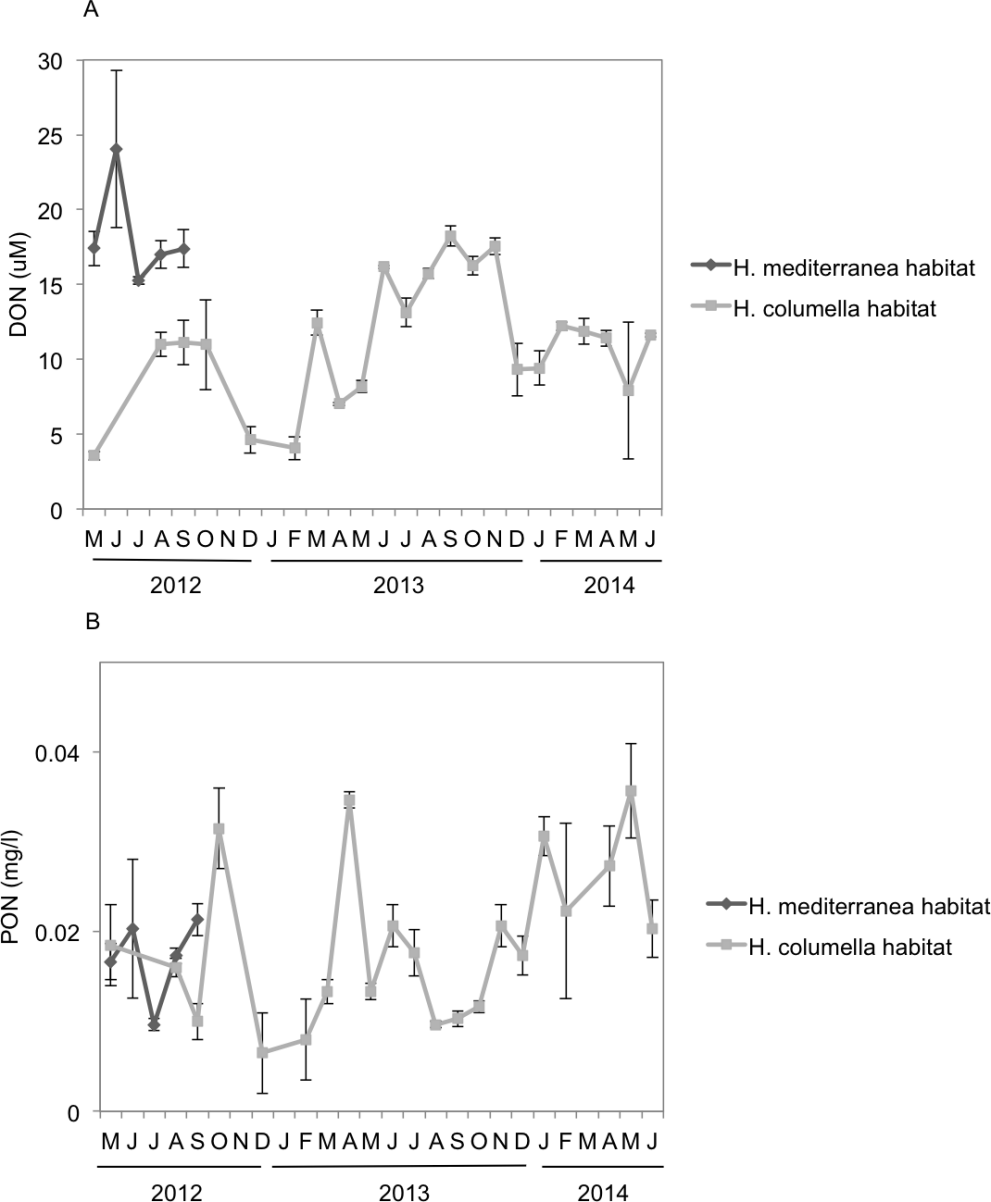
(A) Mean (±SE) concentration of Dissolved Organic Nitrogen (DON). (B) Mean (±SE) concentration of Particulate Organic Nitrogen (PON) *H. columella* and *H. mediterranea* habitats during the entire study period.

Monthly DON values were significantly higher (ANOVA *p* < 0.001) in *H. mediterranea* habitats than in *H. columella* habitats during the period both populations coexisted (2012), with a maximum of ca. 25 µM in July and ca. 11 µM in July to September, respectively (Fig. 7A). During the 21 monitoring months after the *H. mediterranea* population died, DON increased to 17 µM and ca. 12 µM (summer and winter of the second study year, respectively) in the *H. columella* habitat (Fig. 7A).

### Particulate Organic Nitrogen (PON)

Monthly PON values were similar between habitats during the period both species coexisted (ANOVA, *p* = 0.9), ranging from 0.015 µM to 0.022 µM in *H. mediterranea* habitats and from 0.010 µM to 0.030 µM in *H. columella* habitats (Fig. 7B). During the 21 monitoring months after the *H. meditterranea* population died, the highest PON values were 0.035 µM in spring months (April-2013 and May-2014).

### Cross-correlation

**Figure 8:**
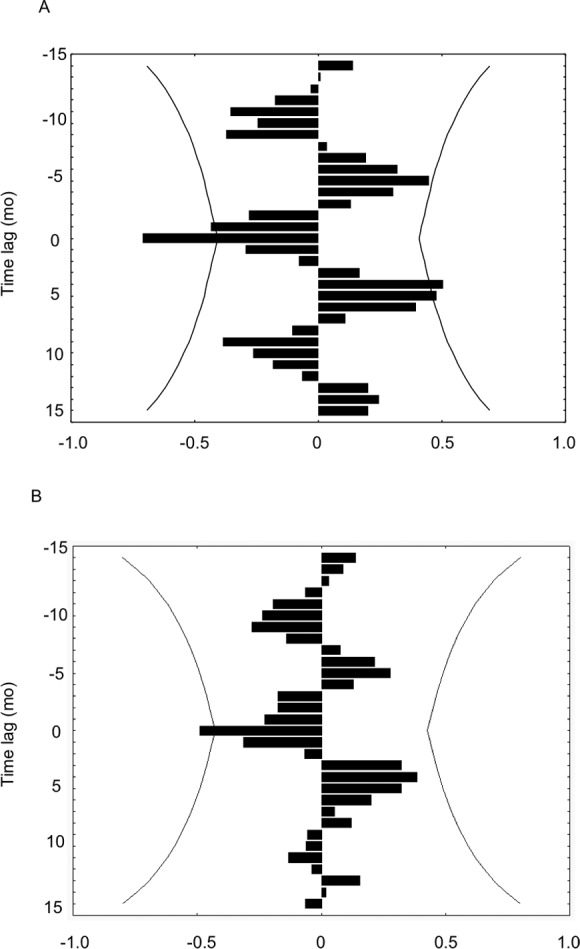
(A) Cross-correlation between the growth rate of *H. columella* and temperature. (B) Cross-correlation between the growth rate of *H. columella* and Dissolved Organic Nitrogen (DON). The left group of bins represents the negative times when the factor growth rate fired prior to the environmental factor. The center or zero bin of the histogram accumulates the number of instances when the two values fired precisely together. The right group of bins accounts for the positive times when the growth values fired posterior to the environmental factor targeted (Weisstein, 2009). Curved lines denote expected 95% confidence intervals

Growth rates of *H. columella* were only correlated, but negatively, with temperature and DON concentration (Figs. 8A and 8B). Growth rates were negatively correlated with the temperature of the same and previous month (time-lag = 0 and −1) (Fig. 8A), and with DON concentration of the same month (time-lag = 0) (Fig. 8B). Conversely, growth rates of *H. mediterranea* were not correlated with any environmental factor analyzed.

## Discussion

*Hemimycale columella* and *H. mediterranea* strongly differed in growth rates during the year. The highest growth rates of *H. columella* occurred in the coldest months and, consequently, the correlation between temperature and the sponge monthly growth rates was negative. Conversely, no correlation between growth rate and temperature was found for *H. mediterranea*. Previous studies have reported contrasting effects of temperature on sponge growth, depending on the species. For example, in the temperate Pacific, the highest growth rate was recorded in winter (10.6 °C in average) for *Latrunculia wellingtonensis,* and in spring (14.5 °C) for *Polymastia croceous,* (Duckworth, Battershill & Schiel, 2004). Positive and negative correlations between temperature and growth have also been reported in both Mediterranean (e.g., Turon, Tarjuelo & Uriz, 1998; Blanquer, Uriz & Agell, 2008; De Caralt, Uriz & Wijffels, 2008; Ferretti et al., 2009) and North Atlantic (Koopmans & Wijffels, 2008) sponge species. Consequently, the potential effect of temperature on sponge growth appears to be species-specific.

Food availability is key for animal growth. Sponges can take dissolved (Yahel et al., 2003; De Goeij et al., 2008) and particulate matter from the surrounding water, and have been reported to be particularly efficient in retaining small particles (Ribes, Coma & Gili, 1999a; Ribes et al., 2005; Lesser, 2006; Jiménez & Ribes, 2007; De Caralt, Uriz & Wijffels, 2008; Koopmans & Wijffels, 2008; Riisgård & Larsen, 2010). Dissolved organic matter was significantly higher in the *H. mediterranea* habitat than in *H*. *columella* habitat, in spring for DON and DOC, and in summer for DON, likely as a result of a higher phytoplankton excretion and decomposer activity in shallow habitats (Ribes, Coma & Gili, 1999b; Pujo-Pay & Conan, 2003). During the period the species coexisted (i.e., spring to autumn), growth rates of *H. mediterranea* were significantly higher than those of *H. columella*. In this period, DOC and DON reached the highest values in *H. mediterranea* habitats, alongside with the higher values of growth rates. However, between-species differences in growth rates could not be compared in winter, as *H. mediterranea* was not present.

*H. mediterranea* does not seem to suffer from the Mediterranean aestivation reported for other filter feeders (Coma & Ribes, 2003) and behaves more alike to other Mediterranean endemisms (e.g., *Scopalina lophyropoda*) that reach their maximum growth rates in summer (Blanquer, Uriz & Agell, 2008). Conversely, growth rates of *H. columella* decreased in summer, and thus the species experiences aestivation, as reported for other sponges, such as *S. blanensis*, with a purported Atlantic origin (Blanquer, Uriz & Agell, 2008).

Fissions and fusions are frequent in encrusting sponge species (Turon, Tarjuelo & Uriz, 1998; Tanaka, 2002; Blanquer, Uriz & Agell, 2008; De Caralt, Uriz & Wijffels, 2008) and have been interpreted as the result of stressing interactions with other organisms such as those involved in competition for space (Pawlik, 1997; Pawlik, 1998; Wulff, 1997; Cebrian & Uriz, 2006), predation, or partial mortality (e.g., Tanaka, 2002). Fusions and fissions were similarly moderate for both species (20% in *H. mediterranea* individuals and ca. 25% in *H. columella*) and lower than those reported for other Mediterranean sponge species: up to 40% and 60% individuals experienced fissions and fusions, respectively in *S. blanensis* (Blanquer, Uriz & Agell, 2008). The highest number of fissions in *H. mediterranea* was recorded after release of larvae, immediately before species demise, possibly as a result of post-reproduction stress (Ereskovsky, 2000). Conversely, no fissions occurred after larval release in *H. columella* which did not experience mortality.

The most striking difference between the two species was their contrasting life spans. The mass death of *H. mediterranea* occurred in early autumn after larval release (Pérez-Porro, González & Uriz, 2012; this study), while 70% of the monitored individuals of *H. columella* persisted after the reproduction period and 64% were still alive after two years. Demise of *H. mediterranea* occurred after release of larvae when sponges have been reported to stop filtering by closing their inhalant orifices (Turon, Uriz & Willenz, 1999), and to devote their energy to the rearrangement of the aquiferous system.

Water flow can also influence sponge success not only by facilitating filtering and thus food availability and excretion of waste materials, but also by either promoting or preventing larval settlement (Maldonado & Uriz, 1998; Uriz, Turon & Mariani, 2008). Settlement of the poorly swimming, sponge larvae has been reported to be more successful on horizontal structurally complex surfaces (Maldonado & Uriz, 1998), such as those at the *H. columella* habitat, than on less complex, shallow rocky boulders, inhabited by *H. mediterranea*. The high individual survival rates recorded, together with a higher potential recruitment rates on the coralligenous assemblages (Maldonado & Uriz, 1998) may have contributed to the persistence of *H. columella* as a consequence of the overlapping of generations.

To summarize, we confirm that the two cryptic sponge species *H. columella* and *H. mediterranea* show contrasting life histories, being *H. columella* multiannual and *H. mediterranea* annual, as it has also been reported to disappear after reproduction in other shallow locations (Pérez-Porro, González & Uriz, 2012). Annual life spans are common in calcareous sponges (Guardiola, Frotscher & Uriz, 2012) while they are rare among Demosponges. Sponge life histories appear to be more diverse than currently though. The strong biological differences showed by these sponge species contrast with their slight differences in phenotypic characters and highlight the need of untangling the cryptic diversity of ecosystems to guarantee the reliability of ecological studies.

##  Supplemental Information

10.7717/peerj.3490/supp-1 Table S1 Raw data of *H. columella* growth rates and mean area throughout the period of study

10.7717/peerj.3490/supp-2 Table S2 Raw data of *H. mediterranea* growth rates and mean area throughout the period of study

10.7717/peerj.3490/supp-3 Table S3 Raw data of environmental factors measured in the *H. columella* habitat

10.7717/peerj.3490/supp-4 Table S4 Raw data of environmental factors measured in the *H. mediterranea* habitat

## Additional Information and Declarations

### Data Availability

The following information was supplied regarding data availability: The raw data has been supplied as Tables S1–S4.
