# Supplementary material for: Contrasting biological features in morphologically cryptic Mediterranean sponges
Source: PeerJ. 2017 Jun 29;5:e3490. doi: 10.7717/peerj.3490 (PMC5493970; doi:10.7717/peerj.3490)
Supplement: Table S1 [file peerj-05-3490-s001.pdf]

## Growth rate

| | MONTHS | IND. 1 | IND. 2 | IND. 3 | IND.4 | IND. 5 | IND. 5N | IND. 5-5 | IND. 6 | IND. 8 | IND. 9 | IND. 11 | IND. 15 | IND. 16 | IND. 18 | IND. 22 |
|---|---|---|---|---|---|---|---|---|---|---|---|---|---|---|---|---|
| JUNE | 1->2 | 0.10792891 | 0.11362372 | | -0.0757152 | -0.1064279 | | | 0.13175698 | | | -0.010853 | | | | |
| JULY | 2->3 | -0.1026214 | -0.282832 | | 0.01518164 | -0.2822756 | | | | -0.1264575 | 0.04311421 | -0.4237557 | -0.0202607 | -0.3336932 | | |
| AUGUST | 3->4 | -0.0698981 | 0.28720247 | | 0.21532847 | 0.63237665 | | | -0.305728 | 0.35734466 | -0.0543208 | 0.53338195 | | | -0.3397784 | 0.41684757 |
| SETEMBER | 4->5 | -0.0294733 | -0.2481532 | -0.0790577 | 0.07782172 | -0.8422758 | | -0.1135285 | | | -0.4525045 | | | | | 0.00792726 |
| OCTOBER | 5->6 | 0.0172176 | -0.7146567 | | -0.7142129 | | | 0.14374619 | | | -0.5851923 | | | | 0.06 | -0.0363835 |
| NOVENMBER | 6->7 | 0.01273732 | | 0.33857455 | 1.49946499 | | 0.48866415 | -0.2055865 | | | | | | | | -0.2515775 |
| DECEMBER | 7->8 | -0.0075978 | | | 0.23307806 | | | | | | | | | | | |
| FEBRUARY | 8->9 | 0.15865083 | | | | | | -0.0453152 | | | | | | | | 0.17316716 |
| APRIL | 9->10 | -0.0066945 | | | 0.21002585 | | 0.10467987 | | | | | | | | | 0.14345101 |
| MAY | 10->11 | 0.14342839 | | | 0.20594956 | | 0.05150102 | | | | | | 0.32843659 | 0.91803943 | | -0.0955351 |
| JUNE | 11->12 | -0.0284223 | | 0.0801419 | 0.09008257 | | 0.20802727 | | | | | | | | | 0.24372853 |
| JULY | 12>13 | -0.0790028 | | | 0.00814901 | | 0.05103024 | | | | | | | | | -0.2372683 |
| AUGUST | 13>14 | -0.1991172 | | -0.1927465 | -0.0522003 | | -0.0321792 | | | | | | 0.01809344 | 0.01125612 | | 0.11252537 |
| SETEMBER | 14>15 | -0.1137999 | | | -0.1665777 | | -0.2645944 | | | | | | | -0.8353006 | | -0.3207663 |
| OCTOBER | 15>16 | -0.1886795 | | -0.226566 | -0.2432865 | | -0.1627814 | | | | | | | -0.4195684 | | 0.02414056 |
| NOVENMBER | 16>17 | -0.1068791 | | 0.08901463 | -0.2629935 | | -0.0015021 | | | | | | | 0.85397933 | | -0.1357224 |
| DECEMBER | 17>18 | -0.2056644 | | 0.14092004 | 0.115835 | | -0.1130109 | -0.0353603 | | | | | | 0.27457652 | | 0.206 |
| JANUARY | 18>19 | -0.17572 | | 0.08052871 | 0.532951 | | 0.44159014 | -0.0143257 | | | | | | 0.45464872 | | 0.218 |
| FEBRUARY | 19>20 | -0.0624881 | | 0.14679276 | 0.232671 | | 0.01850842 | -0.0055761 | | | | | | 0.2496486 | | 0.097 |
| MARCH | 20>21 | -0.1765469 | | 0.0276981 | 0.00407 | | 0.14340452 | 0.13016424 | | | | | | 0.10863588 | | 0.136 |
| APRIL | 21>22 | 0.15685488 | | -0.0281727 | 0.071539 | | 0.0313424 | -0.0433099 | | | | | | 0.04526111 | | 0.258 |
| MAY | 22>23 | -0.3588621 | | -0.1943098 | 0.088384 | | -0.1210579 | -0.2223709 | | | | | | -0.0517943 | | -0.132 |
| JUNE | 23>24 | -0.0827021 | | -0.2419517 | 0.132673 | | -0.0412352 | -0.1062777 | | | | | | -0.0829163 | | -0.15 |

| IND. 23 | IND. 23-2 | IND. 24-2 | IND. 31 | IND. 32 | IND. 33 | IND. 34 | IND. 35 | IND. 36 | IND. 37 | IND. 38 | IND. 39 | MEAN | DESVEST | E.S |
|---|---|---|---|---|---|---|---|---|---|---|---|---|---|---|
| | | | | | | | | | | | | 0.02671893 | 0.1047024 | 0.04274457 |
| | | | | | | | | | | | | -0.1681778 | 0.1677282 | 0.0559094 |
| -0.2795058 | | | | | | | | | | | | 0.12665915 | 0.35115755 | 0.10587799 |
| -0.0208568 | | | | | | | | | | | | -0.1889001 | 0.29180071 | 0.0972669 |
| 0.23295916 | 0.66535079 | | -0.3192744 | | | | | | | | | -0.1250446 | 0.45113484 | 0.14266136 |
| | -0.038607 | -0.3712232 | | | | | | | | | | 0.18405585 | 0.60660939 | 0.21446881 |
| | 0.18675441 | 0.0515404 | 0.07948612 | | | | | | | | | 0.10865223 | 0.09900536 | 0.04427654 |
| 0.14184642 | 0.21394562 | 0.2544684 | 0.13264288 | | | | | | | | | 0.14705801 | 0.09492931 | 0.03587991 |
| | 0.07559966 | 0.27228261 | 0.00336362 | | | | | | | | | 0.11467258 | 0.10288147 | 0.03888554 |
| | 0.18214536 | 0.07754157 | 0.03667307 | | | | | | | | | 0.20535332 | 0.29292445 | 0.09764148 |
| | 0.17667484 | 0.14597105 | 0.235356 | | 0.07004238 | -0.2495845 | -0.2957211 | -0.3992035 | 0.17353549 | -0.0901049 | | 0.0257517 | 0.20919267 | 0.05590909 |
| | -0.1094779 | -0.1046765 | -0.0819871 | 1.05114534 | -0.0549302 | 0.09585333 | 0.21107111 | 0.66959201 | -0.0557317 | -0.1083157 | -0.0128654 | 0.08283903 | 0.3398485 | 0.08774851 |
| | 0.01363172 | 0.11055117 | 0.26239092 | -0.04987 | -0.6667034 | -0.3038401 | 0.17316983 | -0.0997595 | 0.01355806 | -0.0608653 | 0.14891021 | -0.0440664 | 0.20938006 | 0.04935135 |
| | -0.0939451 | -0.3205445 | -0.0692918 | -0.1398318 | -0.3216413 | -0.3743548 | -0.4874881 | -0.2437418 | -0.0094496 | -0.0103294 | -0.1995559 | -0.2482008 | 0.20692435 | 0.05173109 |
| | -0.0807633 | 0.16827301 | -0.0185386 | -0.0601955 | -0.0334146 | 1.1771075 | 0.44932015 | 0.02587269 | -0.1138574 | -0.0940271 | -0.4561975 | -0.0148919 | 0.3711007 | 0.09000514 |
| | -0.3533712 | 0.22332818 | 0.0413978 | 0.41175733 | 0.86853394 | 0.21219657 | -0.0396588 | -0.1283427 | 0.29248515 | -0.0075443 | 0.18438563 | 0.12594496 | 0.33999908 | 0.08246189 |
| | -0.0481293 | 0.226 | 0.067 | 0.06374856 | 0.14382174 | 0.13421901 | -0.1407638 | 0.24974741 | 1.89415328 | | 0.26544944 | 0.19050249 | 0.46311356 | 0.11232154 |
| | 0.09353986 | 0.282 | 0.099 | -0.2088306 | -0.0109799 | 0.38868315 | 0.07080103 | -0.220638 | -0.2899084 | -0.146949 | 0.13333333 | 0.09598468 | 0.25146551 | 0.05927099 |
| | -0.2120086 | 0.251 | -0.2640782 | -0.3006646 | -0.0422586 | 0.21458695 | 0.05357143 | 0.11307054 | 0.19346264 | -0.1507686 | 0.2182107 | 0.04170446 | 0.18145446 | 0.04276923 |
| | -0.0280589 | -0.016 | 0.10212607 | -0.3335345 | 0.05658731 | 0.24305556 | 0.19651855 | -0.0014403 | 0.12150481 | 0.18353129 | 0.06838401 | 0.05367221 | 0.13819141 | 0.03257203 |
| | 0.15228335 | 0.086 | 0.01 | 0.24060661 | -0.342692 | 0.0932468 | -0.0999234 | 0.06193789 | -0.0517519 | 0.00641706 | 0.08248336 | 0.04056236 | 0.13513057 | 0.03185058 |
| | -0.0432645 | -0.04 | 0.095 | -0.3110746 | 0.17713568 | -0.0611332 | -0.2854105 | 0.01342282 | -0.2134326 | -0.2479505 | -0.1134247 | -0.1123413 | 0.14897816 | 0.03511449 |
| | -0.2594599 | 0.018 | -0.099 | 0.02648305 | -0.0576307 | 0.00992516 | -0.1547619 | 0.06228319 | 0.03727625 | 0.0314273 | 0.11669803 | -0.0467316 | 0.11152293 | 0.02628621 |

## Area (cm2)

| | MONTHS | IND. 1 | IND. 2 | IND. 3 | IND.4 | IND. 5 | IND. 5 N | IND. 5-5 | IND. 6 | IND. 8 | IND. 9 | IND. 11 | IND. 15 | IND. 16 | IND. 18 | IND. 22 |
|---|---|---|---|---|---|---|---|---|---|---|---|---|---|---|---|---|
| MAY | 1 | 109.044 | 41.347 | 12.045 | 23.945 | 24.176 | | | 15.225 | 117.494 | | 21.653 | | | | |
| JUNE | 2 | 120.813 | 46.045 | | 22.132 | 21.603 | | | 17.231 | | 71.299 | 21.418 | 17.028 | 11.115 | | |
| JULY | 3 | 108.415 | 33.022 | | 22.468 | 15.505 | | | | 87.778 | 74.373 | 12.342 | 16.683 | 7.406 | 9.206 | 21.19 |
| AUGUST | 4 | 100.837 | 42.506 | | 27.306 | 25.31 | | 61.086 | 6.695 | 119.145 | 70.333 | 18.925 | | | | 30.023 |
| SETEMBER | 5 | 97.865 | 31.958 | 8.236 | 29.431 | 3.992 | | 54.151 | | | 38.507 | | | | 2.95 | 30.261 |
| OCTOBER | 6 | 99.55 | 9.119 | | 8.411 | | 15.129 | 61.935 | | | 15.973 | | | | 3.127 | 29.16 |
| NOVENMBER | 7 | 100.818 | | 13.813 | 21.023 | | 22.522 | 49.202 | | | | | | | | 21.824 |
| DECEMBER | 8 | 100.052 | | | 25.923 | | | | | | | | | | | |
| FEBRUARY | 9 | 123.862 | | | | | | 43.628 | | | | | | | | 31.272 |
| APRIL | 10 | 121.789 | | | 47.701 | | 34.31 | | | | | | | | | 42.487 |
| MAY | 11 | 139.257 | | | 57.525 | | 36.077 | | | | | | 87.914 | 75.396 | | 38.428 |
| JUNE | 12 | 135.299 | | 21.562 | 62.707 | | 43.582 | | | | | | | | | 47.794 |
| JULY | 13 | 124.61 | | | 63.218 | | 45.806 | | | | | | | | | 36.454 |
| AUGUST | 14 | 99.798 | | 13.25 | 59.918 | | 44.332 | | | | | | 92.686 | 77.942 | | 40.556 |
| SETEMBER | 15 | 88.441 | | | 49.937 | | 32.602 | | | | | | | 12.837 | | 27.547 |
| OCTOBER | 16 | 71.754 | | 7.246 | 37.788 | | 27.295 | | | | | | | 7.451 | | 28.212 |
| NOVENMBER | 17 | 64.085 | | 7.891 | 27.85 | | 27.254 | | | | | | | 13.814 | | 24.383 |
| DECEMBER | 18 | 50.905 | | 9.003 | 31.076 | | 24.174 | 28.201 | | | | | | 17.607 | | 29.406 |
| JANUARY | 19 | 41.96 | | 9.728 | 47.638 | | 34.849 | 27.797 | | | | | | 25.612 | | 35.809 |
| FEBRUARY | 20 | 39.338 | | 11.156 | 58.722 | | 35.494 | 27.642 | | | | | | 32.006 | | 39.281 |
| MARCH | 21 | 32.393 | | 11.465 | 58.961 | | 40.584 | 31.24 | | | | | | 35.483 | | 44.609 |
| APRIL | 22 | 37.474 | | 11.142 | 63.179 | | 41.856 | 29.887 | | | | | | 37.089 | | 56.13 |
| MAY | 23 | 24.026 | | 8.977 | 68.763 | | 36.789 | 23.241 | | | | | | 35.168 | | 48.743 |
| JUNE | 24 | 22.039 | | 6.805 | 77.886 | | 35.272 | 20.771 | | | | | | 32.252 | | 41.422 |

| IND. 23 | IND. 23-2 | IND. 24-2 | IND. 31 | IND. 32 | IND. 33 | IND. 34 | IND. 35 | IND. 36 | IND. 37 | IND. 38 | IND. 39 | Mean | desvest | s.e |
|---|---|---|---|---|---|---|---|---|---|---|---|---|---|---|
| | | | | | | | | | | | | 45.616125 | 42.6981742 | 15.0960843 |
| | | | | | | | | | | | | 38.7426667 | 36.0835342 | 12.0278447 |
| 20.962 | | | | | | | | | | | | 35.7791667 | 34.2752021 | 9.89439857 |
| 15.103 | | | | | | | | | | | | 47.0244545 | 36.6647008 | 11.0548232 |
| 14.788 | 34.451 | | 92.278 | | | | | | | | | 36.5723333 | 31.2740567 | 9.02804252 |
| 18.233 | 57.373 | 32.832 | 62.816 | | | | | | | | | 34.4715 | 29.5921314 | 8.54251252 |
| | 55.158 | 20.644 | | | | | | | | | | 38.1255 | 29.3781955 | 10.3867606 |
| | 65.459 | 21.708 | 72.802 | | | | | | | | | 57.1888 | 33.1129112 | 14.8085441 |
| 27.285 | 86.466 | 27.232 | 87.287 | | | | | | | | | 61.0045714 | 38.1961469 | 14.4367865 |
| | 102.808 | 45.769 | 88.021 | | | | | | | | | 68.9835714 | 34.6196427 | 13.084995 |
| | 121.534 | 49.318 | 91.249 | | 17.932 | 10.83 | 3.155 | 17.828 | 14.51 | 43.072 | | 53.6016667 | 41.5398085 | 10.7255324 |
| | 143.006 | 56.517 | 112.725 | 5.064 | 19.188 | 8.127 | 2.222 | 10.711 | 17.028 | 39.191 | 54.798 | 48.7200625 | 45.2167431 | 11.3041858 |
| | 127.35 | 50.601 | 103.483 | 10.387 | 18.134 | 8.906 | 2.691 | 17.883 | 16.079 | 34.946 | 54.093 | 47.6427333 | 41.0921589 | 10.6099498 |
| | 129.086 | 56.195 | 130.636 | 9.869 | 6.044 | 6.2 | 3.157 | 16.099 | 16.297 | 32.819 | 62.148 | 49.8351111 | 41.8898052 | 9.87352178 |
| | 116.959 | 38.182 | 121.584 | 8.489 | 4.1 | 3.879 | 1.618 | 12.175 | 16.143 | 32.48 | 49.746 | 38.5449375 | 38.7260092 | 9.68150231 |
| | 107.513 | 44.607 | 119.33 | 7.978 | 3.963 | 8.445 | 2.345 | 12.49 | 14.305 | 29.426 | 24.73 | 32.6398824 | 35.2642858 | 8.55284559 |
| | 69.521 | 54.569 | 124.27 | 11.263 | 7.405 | 10.237 | 2.252 | 10.887 | 18.489 | 29.204 | 32.131 | 31.5002941 | 31.1202607 | 7.54777189 |
| | 66.175 | 66.886 | 132.579 | 11.981 | 8.47 | 11.611 | 1.935 | 13.606 | 53.51 | | 40.545 | 35.1570588 | 32.2649321 | 7.82539546 |
| | 72.365 | 85.757 | 145.722 | 9.479 | 8.377 | 16.124 | 2.072 | 10.604 | 37.997 | 20.621 | 45.951 | 37.6923333 | 34.9960673 | 8.24865217 |
| | 57.023 | 107.242 | 107.24 | 6.629 | 8.023 | 19.584 | 2.183 | 11.803 | 45.348 | 17.512 | 55.978 | 37.9002222 | 30.9090371 | 7.28532992 |
| | 55.423 | 105.498 | 118.192 | 4.418 | 8.477 | 24.344 | 2.612 | 11.786 | 50.858 | 20.726 | 59.806 | 39.8263889 | 32.1504753 | 7.57793971 |
| | 63.863 | 114.581 | 119.362 | 5.481 | 5.572 | 26.614 | 2.351 | 12.516 | 48.226 | 20.859 | 64.739 | 42.2733889 | 34.1818487 | 8.05673901 |
| | 61.1 | 109.945 | 130.691 | 3.776 | 6.559 | 24.987 | 1.68 | 12.684 | 37.933 | 15.687 | 57.396 | 39.3413889 | 35.7761844 | 8.43252753 |
| | 45.247 | 111.9 | 117.704 | 3.876 | 6.181 | 25.235 | 1.42 | 13.474 | 39.347 | 16.18 | 64.094 | 37.8391667 | 34.7021627 | 8.17937819 |
