# Supplementary material for: Contrasting biological features in morphologically cryptic Mediterranean sponges
Source: PeerJ. 2017 Jun 29;5:e3490. doi: 10.7717/peerj.3490 (PMC5493970; doi:10.7717/peerj.3490)
Supplement: Table S2 [file peerj-05-3490-s002.pdf]

Growth rate

	MONTHS	IND.1.1	IND. 1.2	IND. 2	IND. 3	IND. 4	IND. 5	IND. 6	IND. 7	IND. 8	IND. 9	IND. 10	IND. 11-1	IND. 11-2
MARCH	1->2	0.07135969	-0.0270822	-0.23956	-0.1085155	0.04669149	0.2159187	-0.1544168	-0.21843	0.03120509		-0.0241614	-0.1569727	-0.5932978
MAY	2->3	0.46534653	0.33289149	-0.313044	0.41619736	0.48166393	0.03453294	0.52007122	0.85868164		0.0427718	0.16891556	-0.074813	-0.3321659
JUNE	3->4	0.75614251	-0.0621271	0.42811889	0.16771591	-0.0157985	0.22249002	0.15450964	0.05050121	-0.0013438	0.12566036	-0.0615502	0.10062893	-0.7234336
JULY	4->5	-0.7667016	-0.163736	-0.2684629	0.035515	0.02967708	-0.1130281	-0.0592695	-0.1440499	-0.2437369	-0.0376916	-0.1367393	0.32897959	0.33233533
AUGUST	5->6	4.8035982	0.21682164	0.16801926	0.5646396	0.21561623	0.26659671	0.16118595	0.09189768	-0.189323	-0.1919785	-0.2038569	1.24078624	0.6
SEPTEMBER	6->7		0.17216626	-0.2289837	0.02566284	0.02990684	0.03882625	0.20923046	0.00938946	0.55107855	0.06538619	-0.7057863		

IND. 11-2,2	IND. 11-3	IND. 11-4	IND. 12	IND. 14	IND. 15	IND. 16	IND. 17	IND. 20	IND. 21	IND. 21-E	MEAN	DESVEST.	E.ST.
	-0.1754084	-0.2856746	0.26396355		-0.1139264	0.00904393		0.16856939			-0.0717052	0.20357536	0.04798317
	-0.4014599	0.33942813	0.42564637	0.75435988	0.24531239	0.32210655	0.19988131	0.42069913	0.68474216		0.26627455	0.344477	0.07517104
	0.82229965	-0.0743538	0.18186847	0.32996841	-0.1100488	-0.0861089	0.07749704	-0.0463092		-0.0111609	0.10114392	0.31188082	0.06649322
-0.1128527		0.2828679	-0.4645717	0.01402167	0.1198355	0.11790453	0.27921132	0.11446598	0.05936415	-0.0944496	-0.038744	0.25496084	0.05316301
	0.18594646	0.48037177	0.58425949	0.20610251	0.2001808	0.44252566	0.47034321		0.10753826	0.60306807	0.50110634	1.01398781	0.21618293
			0.04027824	0.1440686	-0.0259203	-0.1130857	-0.1063884		-0.3307016	0.03759398	-0.0110164	0.2611236	0.06333178

Area (cm2)

		IND.1.1	IND. 1.2	IND. 2	IND. 3	IND. 4	IND. 5	IND. 6	IND. 7	IND. 8	IND. 9	IND. 10	IND. 11-1	IND.11-2,1	IND. 11-2,2	IND. 11-3
FEBRUARY	1	1.037	27.915	20.546	82.661	52.365	76.853	36.531	30.765	19.484	33.48	39.112	1.427	13.339		1.163
MARCH	2	1.111	27.159	15.624	73.691	54.81	93.447	30.89	24.045	20.092		38.167	3.442	3.234		1.004
MAY	3	1.628	36.2	10.733	104.361	81.21	96.674	46.955	44.692		36.344	44.614	1.113	3.623		0.574
JUNE	4	2.859	33.951	15.328	121.864	79.927	118.183	54.21	46.949	20.038	40.911	41.868	1.225	1.002	1.914	1.046
JULY	5	0.667	28.392	11.213	126.192	82.299	104.825	50.997	40.186	15.154	39.369	36.143	1.628	1.335	1.698	
AUGUST	6	4.312	34.548	13.097	197.445	100.044	132.771	59.217	43.879	12.285	31.811	28.775	3.648	2.136		1.435
SEPTEMBER	7	4.95	40.496	10.098	202.512	103.036	137.926	71.607	44.291	19.055	33.891	8.466				

IND. 11-4	IND. 12	IND. 12	IND. 14	IND. 14	IND. 15	IND. 16	IND. 16	IND. 17	IND. 17	IND. 20	IND. 21	IND. 21-E	Area Mean	desvest	s.e
7.197	28.091				80.473	17.802				5.606			30.3077368	26.2349476	6.01870976
5.401	35.506		7.397		71.305	17.963		38.758		6.551	7.524		27.4819524	26.5130201	5.78561532
6.886	50.619			12.977	88.797	23.749		46.505		9.307	12.676	16.307	35.2974545	32.5733618	6.94466406
6.374		59.825		17.259	79.025	21.704		50.109		8.876		16.125	36.5466087	35.8936115	7.48433536
8.177	32.032			17.501	88.495	24.263			64.1	9.892	14.181	14.602	35.3626522	35.4229337	7.38619226
12.105	50.747			21.108	106.21		35		94.249		15.706	23.408	46.5425455	50.3046757	10.7249929
	52.791			24.149	103.457		31.042		84.222		10.512	24.288	55.9327222	52.9197248	12.4732988
