# Supplementary material for: Contrasting biological features in morphologically cryptic Mediterranean sponges
Source: PeerJ. 2017 Jun 29;5:e3490. doi: 10.7717/peerj.3490 (PMC5493970; doi:10.7717/peerj.3490)
Supplement: Table S3 [file peerj-05-3490-s003.pdf]

	Temperature (ºC)		DOC (uM)		POC (mg/l)		DON (uM)		PON (mg/l)	
	Mean value	S.E	Mean value	S.E	Mean value	S.E	Mean value	S.E	Mean value	S.E
M	15.2835714		66.06	0.03	0.208	0.029	3.56	0.3	0.0185	0.0045
J	15.4642857	0.04274457								
J	20.215	0.0559094								
A	18.0282143	0.10587799	81.595	6.115	0.824	0.033	11	0.8	0.016	0.001
S	19.4421429	0.0972669	99.19	9.53	0.5875	0.1435	11.11	1.49	0.01	0.002
O	18.3478571	0.14266136	96.52	0	0.2615	0.0985	10.982	3.02	0.0315	0.0045
N	16.1	0.21446881								
D	14.0575	0.04427654			0.0735	0.0045	4.627	0.885	0.0065	0.0045
J										
F	12.9975	0.03587991	95.685	3.525	0.0625	0.0105	4.079	0.776	0.008	0.0045
M			66.83	0	0.118	0.026	12.4506667	0.8526305	0.01333333	0.00133333
A	13.1310714	0.03888554	81.7633333	1.70616855	0.34733333	0.00578312	7.02066667	0.10941105	0.03466667	0.00088192
M	14.0035714	0.09764148	84.6166667	1.1209272	0.13666667	0.00375648	8.19	0.42003968	0.01333333	0.00088192
J	17.695	0.05590909	74.5466667	0.60454758	0.19566667	0.04387989	16.18	0.10583005	0.02066667	0.00233333
J	15.5842857	0.08774851	62.676	1.43604538	0.18666667	0.02750959	13.1366667	0.94809165	0.01766667	0.00260342
A	20.1310714	0.04935135	75.6976667	1.62159688	0.082	0.00404145	15.7233333	0.34844097	0.00966667	0.00033333
S	19.3717857	0.05173109	68.43	0.67300322	0.09766667	0.01266667	18.25	0.67470981	0.01033333	0.00088192
O	18.1963571	0.09088414	81.975	3.14759432	0.114	0.004	16.2586667	0.63024687	0.01166667	0.00066667
N	16.465	0.08303615	64.7266667	1.55201303	0.243	0.08411302	17.5653333	0.57684005	0.02066667	0.00233333
D	13.3292857	0.11232154	63.6833333	0.98786526	0.187	0.02655811	9.31	1.74646309	0.01733333	0.00218581
J	13.7010714	0.05927099	78.89	6.65902646	0.49266667	0.05011431	9.41666667	1.12836657	0.03066667	0.00218581
F	13.38	0.04276923	68.3333333	2.0174599	0.19133333	0.02544493	12.2233333	0.29036376	0.02233333	0.00976957
M	13.8457143	0.03257203	83.4866667	2.84130447	0.72133333	0.26529062	11.8733333	0.85658884		
A	14.4610714	0.03185058	72.3033333	2.0348983	0.30033333	0.06165045	11.4166667	0.53345832	0.02733333	0.00448454
M	15.6032143	0.03511449	73.9566667	2.67195517	0.41433333	0.08883755	7.91333333	4.56174796	0.03566667	0.00523874
J	16.3221875	0.02628621	71.7566667	1.21084452	0.22066667	0.04442347	11.6066667	0.11551816	0.02033333	0.0031798
