# Supplementary material for: Contrasting biological features in morphologically cryptic Mediterranean sponges
Source: PeerJ. 2017 Jun 29;5:e3490. doi: 10.7717/peerj.3490 (PMC5493970; doi:10.7717/peerj.3490)
Supplement: Table S4 [file peerj-05-3490-s004.pdf]

	Temperature (ºC)		DOC (uM)		POC (mg/l)		DON (uM)		PON (mg/l)	
			Mean value	S.E	Mean value	S.E	Mean value	S.E	Mean value	S.E
F	12.6192857									
M	13.5432143	0.04798317								
A	14.005		83.2566667	1.20277919	0.14066667	0.00272845	4.62033333	0.51836549	0.014	0.00057735
M	13.77	0.07517104	80.4166667	2.55072757	0.137	0.01301281	17.41	1.15052162	0.01666667	0.00202759
J	19.6945833	0.06649322	115.566667	23.5898342	0.195	0.02364318	24.0566667	5.2512517	0.02033333	0.00775314
J	21.2282143	0.05316301	69.4883333	3.68319573	0.096	0.00450925	15.27	0.27622455	0.00966667	0.00066667
A	23.6664286	0.21618293	78.22	3.17579911	0.216	0.01646208	17.012	0.9266247	0.01733333	0.00088192
S	19.4642857	0.06333178	69.3466667	1.49584239	0.34966667	0.03464262	17.3953333	1.24816309	0.02133333	0.00176383
